# Accumulation of Senescent Neural Cells in Murine Lupus With Depression-Like Behavior

**DOI:** 10.3389/fimmu.2021.692321

**Published:** 2021-11-03

**Authors:** Yuki Saito, Maki Miyajima, Sena Yamamoto, Tsukasa Sato, Norihiro Miura, Mineko Fujimiya, Takako S. Chikenji

**Affiliations:** ^1^ Department of Anatomy, Sapporo Medical University School of Medicine, Sapporo, Japan; ^2^ Department of Health Sciences, School of medicine, Hokkaido University, Sapporo, Japan

**Keywords:** systemic lupus erythematosus, senescence, depression, inflammation, SASP (senescence-associated secretory phenotype)

## Abstract

Neuropsychiatric manifestations targeting the central, peripheral, and autonomic nervous system are common in systemic lupus erythematosus (SLE); collectively, these symptoms are termed neuropsychiatric SLE (NPSLE). Among a wide variety of neuropsychiatric symptoms, depression is observed in about 24-39% of SLE patients. Several cytokines and chemokines have been identified as biomarkers or therapeutic targets of NPSLE; in particular, the levels of type 1 interferons, TNFs, and IL-6 are elevated in SLE patient’s cerebrospinal fluid (CSF), and these factors contribute to the pathology of depression. Here, we show that senescent neural cells accumulate in the hippocampal cornu ammonis 3 (CA3) region in MRL/lpr SLE model mice with depressive behavior. Furthermore, oral administration of fisetin, a senolytic drug, reduced the number of senescent neural cells and reduced depressive behavior in the MRL/lpr mice. In addition, transcription of several senescence and senescence-associated secretory phenotype (SASP) factors in the hippocampal region also decreased after fisetin treatment in the MRL/lpr mice. These results indicate that the accumulation of senescent neural cells in the hippocampus plays a role in NPSLE pathogenesis, and therapies targeting senescent cells may represent a candidate approach to treat NPSLE.

## Introduction

Systemic lupus erythematosus (SLE) is a currently incurable autoimmune disease characterized by hyperactive immune cells, serum autoantibodies, and multiple organ damage involving the kidney, skin, vasculature, and brain (1). Neuropsychiatric manifestations targeting the central, peripheral, and autonomic nervous system are common in SLE; collectively, these symptoms are called neuropsychiatric SLE (NPSLE). Up to 75% of patients experience central nervous system (CNS) involvement, and 60% of SLE patients experience autonomic symptoms (2–4). Among a wide variety of neuropsychiatric symptoms, depression is observed in about 24-39% of SLE patients (5). Various immune effectors contribute to SLE pathogenesis, including autoantibodies, cytokines, and cell-mediated inflammation (2, 4, 6, 7); however, the detailed mechanism underlying NPSLE remains largely unknown (2, 4, 6, 7).

Cellular senescence is a state of irreversible cell cycle arrest in which an adaptive response is induced by multiple stressors (8, 9). Although senescence serves as a defense mechanism that limits tumorigenesis to maintain tissue homeostasis, accumulation of senescent cells causes age-related disease and chronic inflammation in lung, kidney, heart, and muscle, through the secretion of pro-inflammatory molecules including cytokines, chemokines, and proteases; collectively, these factors are referred to as the senescence-associated secretory phenotype (SASP) (8–13). Prolonged exposure to the SASP leads to pathological changes that contribute to tissue and organ decline (8). Senescent cells contribute to the neurodegeneration and pathogenesis of the brain observed in Alzheimer’s disease, Parkinson’s disease, and multiple sclerosis (14–18). For example, in Alzheimer’s disease model mice, astrocytes, microglia, and oligodendrocyte progenitor cells have features of senescence, and elimination of senescent cells *via* genetic or pharmacological treatment attenuates neuroinflammation and cognitive deficits (17, 18). Chronic neuroinflammation is one of the hallmarks of Parkinson’s disease. The expression levels of pro-inflammatory factors and proteases, such as tumor necrosis factor-α (TNF-α), interleukin-1β (IL-1β), and interleukin-6 (IL-6), and interferon-gamma (IFN-γ) and metalloproteinase-3 (MMP-3), which are canonical SASP factors, are elevated in the brains of patients with Parkinson’s disease (19). Furthermore, the number of senescent astrocytes and dopaminergic neurons is elevated, and these senescent cells have the potential to contribute to pathology (14, 15). Although the mechanism by which cellular senescence is linked to neurodegeneration is not fully understood, the accumulation of senescent cells may trigger a chronic inflammatory response that contributes to synapse damage and cognitive decline (20). NPSLE causes a disruption of the blood–brain barrier, which is directly caused by cytokines and complement proteins (21). Pro-inflammatory cytokines and chemokines related to neuroinflammation in NPSLE were identified in the cerebrospinal fluid (CSF) of SLE patients for use as biomarkers or therapeutic targets; in particular, the levels of type 1 interferons, TNFs, and IL-6 are elevated and contribute to the pathology of depression (4, 6, 8, 22). Overexpression of these pro-inflammatory factors in the CSF of NPSLE patients is hypothesized to cause cellular senescence in CNS; however, to the best of our knowledge, cellular senescence in the CNS has not been evaluated in patients with lupus (22–30).

In this study, we sought to determine the relationship between senescence and depression in SLE by investigating cellular senescence in the hippocampus, which is associated with depression (31–34), in MRL/*lpr* SLE model mice. In addition, we investigated whether senolytics, small molecules that selectively eliminate senescent cells, reduce the observed number of senescent cells and consequently reduce depression symptoms in MRL/*lpr* SLE model mice.

## Materials and Methods

### Mice 

The Committee of the Animal Experimentation Center of the Sapporo Medical University School of Medicine approved all animal protocols (#17-080 and #21-051). Mice were maintained in an enclosed, specific pathogen–free facility with a 12 h light and dark cycle. Female MRL/*lpr* mice were used as an SLE mouse model, and haplotype-matched female MRL/*MPJ* mice were used as phenotypic controls (Sankyo Lab Service, Tokyo, Japan). For pathological analysis, four MRL/*MPJ* mice and five MRL/*lpr* mice were used and euthanized at 18 weeks of age. For senolytic treatment, twenty-four MRL/*MPJ* mice and twenty-four MRL/*lpr* mice were used and euthanized at 22 weeks of age, and tissue samples were harvested.

### Behavioral Analysis

The tail suspension test was performed to assess depression-like behavior (35–39). Mice were suspended by their tails with tape 60 cm above the floor for 6 min, and the time of immobility was measured. Each mouse was tested only once. The time of immobility was defined as the time when the animal stopped struggling for ≥ 1 s, which was measured using a video tracking system (ANY-maze; Muromachi Kikai, Tokyo, Japan).

### Cell Culture and Senescence Induction

Neuro-2a cells (Cell No. IFO50081), which are a mouse brain–derived neuroblast cell line, were obtained from the Japanese Collection of Research Bioresources Cell Bank (Osaka, Japan) and maintained in Eagle’s Minimal Essential Medium with non-essential amino acids and 10% fetal bovine serum. Cells were tested for mycoplasma using the e-Myco Mycoplasma PCR Detection Kit (iNtRON Biotechnology, Seongnam-si, South Korea). Cellular senescence was induced by X-ray irradiation. Neuro-2a cells were exposed to 10 Gy irradiation using an X-Ray Irradiator (MBR-1520-3; HITACHI, Tokyo, Japan), and 3 days later the cells were passaged to avoid confluency. Six days after irradiation, Neuro-2a cells were harvested and subjected to SA-β-Gal staining, PCR analysis, and pharmacological experiments. To detect cellular senescence, we performed senescence-associated β-Galactosidase (SA-β-Gal) staining using the Senescence β-Galactosidase Staining Kit (Cell Signaling Technology, Danvers, MA, USA). Cells were observed using an inverted microscope (Primovert; ZEISS), and the percentage of SA β-Gal–positive cells was calculated by dividing the number of SA β-Gal–positive cells by the total number of cells observed. The cell size was measured using the ImageJ software (National Institutes of Health). Briefly, the cell body was outlined using the drawing/selection tools, and the area was measured using the analyze tool.

### *In Vitro* Senolytic Treatment

For senolytic treatment, fisetin flavonoid, which is found in many fruits and vegetables and was previously identified as a senolytic compound (40), was used. Fisetin (S2298) was purchased from Selleck (Houston, TX, USA) and dissolved in DMSO before use. Irradiated senescent Neuro-2a cells and non-irradiated Neuro-2a cells were seeded on a 96-well black/clear bottom plate at 40,000 cells and 10,000 cells per well, respectively. After senescence induction for 6 days, 5µM, 10µM, or 20µM fisetin was added and the cells were incubated for 48 h. The concentration of fisetin used was based upon a previous study that reported its senolytic effects (40). Cell number and cellular senescence were determined by DAPI staining and SPiDER-β-Gal staining, respectively. Briefly, cells were washed twice with PBS, fixed in 4% paraformaldehyde at room temperature for 5 min, and washed twice with PBS. Sections were incubated in 20 µM SPiDER-β-gal (Dojindo) in solution in McIlvaine buffer (pH 6.0) for 60 min at 37°C. After washing of tissue sections, nuclei were stained with DAPI. Cells were observed using fluorescence microscopy (Axio Observer7; ZEISS).

### *In Vivo* Senolytic Treatment

Eighteen-week–old MRL/*MPJ* mice (n = 24) and MRL/*lpr* mice (n = 24) were randomized for pharmacological treatment analysis. For oral administration, mice were gavaged with 100 mg/kg fisetin (Tokyo Chemical Industry, Tokyo, Japan) (MRL/*MPJ*: n = 12 and MRL/lpr: n = 12) or vehicle (20% PEG 400) (MRL/*MPJ*: n = 12 and MRL/*lpr*: n = 12) for 5 days every week for 4 weeks.

### Immunohistochemistry and SPiDER-β-Gal Staining

Brain samples were fixed in 4% paraformaldehyde overnight. The following day, the tissues were transferred to 20% sucrose in phosphate buffer, incubated overnight, frozen in OCT compound in liquid nitrogen, and stored at −80°C until use. Cryosections (8 µm thick) were prepared using a cryostat. The sections were incubated in 0.01 M PBS containing 0.3% Triton-X (PBS-T) and treated with 2% BSA for 60 min at RT. After washing with 0.01 M PBS-T, the sections were incubated with primary antibodies at 4°C overnight, followed by secondary staining. Alexa Fluor 594–conjugated anti-GFAP (1:100; 644708; Biolegend), anti–Iba-1 (1:400; 019-19741; Wako, Osaka, Japan), and anti-NeuN (1:150; 2697501; Proteintech, Rosemont, IL, USA) were used as primary antibodies. Cy3-conjugated IgG (1:400; Jackson ImmunoResearch, West Grove, PA, USA) was used as a secondary antibody, and nuclei were stained with DAPI (1:1000; Dojindo). After washing, tissue sections were mounted with Vectashield (Vector Laboratories). For the SPiDER-β-gal stain, tissue sections were incubated in 20 µM SPiDER-β-gal (Dojindo) in solution in McIlvaine buffer (pH 6.0) for 60 min at 37°C. After washing of tissue sections, nuclei were stained with DAPI, and tissue sections were mounted with VECTASHIELD. Sections were observed by fluorescence microscopy [Axio Observer7 (ZEISS) or BZ-X700 (Keyence)].

### RNA Extraction and Quantitative Real-Time PCR

Total RNA was isolated using the Tri Reagent (Molecular Research Center, Cincinnati, OH, USA), and RNA was reverse transcribed into cDNA using the iScript Advanced cDNA Synthesis Kit (1725038; Bio-Rad, Hercules, CA, USA). Quantitative PCR was performed using SsoAdvanced Universal SYBR Green Supermix (172-5270; Bio-Rad) on a QuantStudio3 Real-Time PCR System (Applied Biosystems). Cycling conditions were as follows: 95°C for 20 s, followed by 40 cycles of amplification (95°C for 15 s and 60°C for 1 min). Transcription levels were normalized against the corresponding levels of housekeeping genes listed in **Supplementary Table 1**. Specific primer sequences used for PCR are listed in **Supplementary Table 1**. The ΔΔCt method was used to compare data.

### Statistical Analysis

Quantitative data are shown as means and standard errors in dot plots generated by ggplot2, a plotting system for R based on The Grammar of Graphics (The R Foundation for Statistical Computing, Vienna, Austria). Normality was assessed using the Shapiro–Wilk test. The pairwise t-test was used for comparison between two groups, and a one-way analysis of variance (ANOVA) was conducted to assess differences among three groups or more. Pairwise comparisons were made only when one-way ANOVA indicated statistical significance. P-values for multiple comparisons were adjusted by the Tukey method. Statistical analyses were performed using EZR, a graphical user interface for R (41). Two-sided P-values less than 0.05 were considered statistically significant.

## Results

### MRL/lpr Mice Exhibit a Depression-Like Phenotype and Neuroinflammation in the Hippocampus

**Figure 1:**
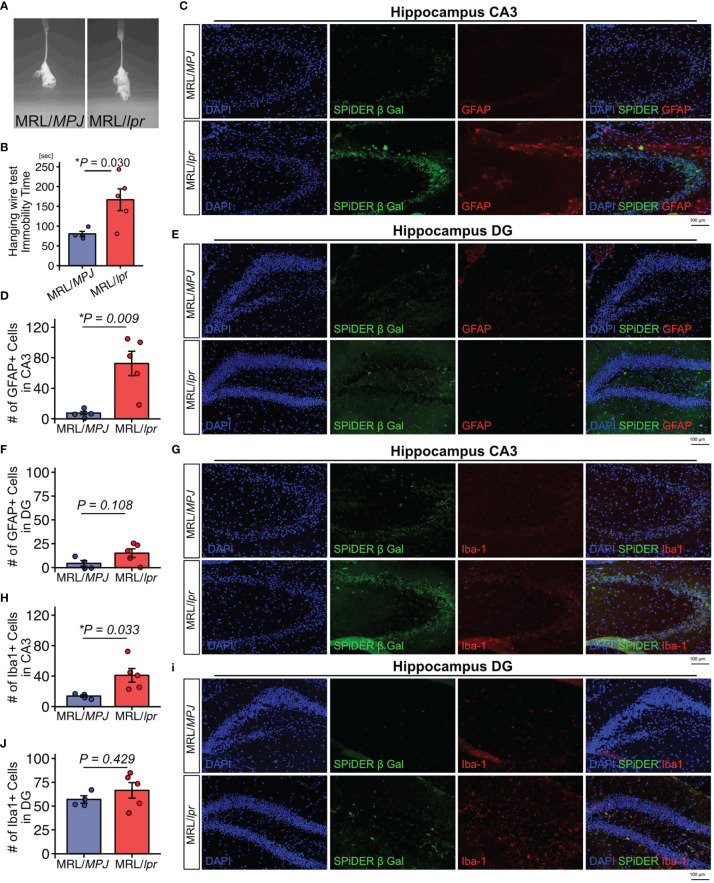
MRL/*lpr* mice exhibit a depression-like phenotype and have higher proportions of GFAP-positive and Iba-1–positive cells in the hippocampus. **(A)** Representative images of tail suspension test of MRL/*lpr* mice (SLE model) and MRL/*MPJ* mice (controls). **(B)** Quantitation of immobility time in MRL/MPJ and MRL/lpr mice during tail suspension test. **(C)** Representative images of GFAP immunostaining of the hippocampus CA3 regions from MRL/*MPJ* and MRL/*lpr* mice and **(D)** the corresponding quantitative data. **(E)** Representative images of GFAP immunostaining of the hippocampus DG regions from MRL/*MPJ* and MRL/*lpr* mice and **(F)** the corresponding quantitative data. **(G)** Representative images of Iba-1 immunostaining of the hippocampus CA3 regions from MRL/*MPJ* and MRL/*lpr* mice and **(H)** the corresponding quantitative data. **(I)** Representative images of Iba-1 immunostaining of the hippocampus DG regions from MRL/*MPJ* and MRL/*lpr* mice and **(J)** the corresponding quantitative data. Quantitative data are shown as means ± SEs in dot plots. P-values were determined by two-tailed Student’s t-test. (*P < 0.05) .

We used 18-week-old MRL/*lpr* mice as a SLE model and MRL/*MPJ* mice as controls. The presence of depression-like behavior was evaluated by tail suspension test. The immobility time observed during the tail suspension test was significantly elevated in MRL/*lpr* mice relative to the MRL/MPJ mice, which indicated that the MRL/*lpr* mice exhibited a depression-like phenotype (**Figures 1A, B**). To determine whether MRL/*lpr* mice exhibited neuroinflammation, we counted GFAP-positive astrocytes and Iba-1–positive microglia in the hippocampus, which are regions that may be important in regulation of emotion in brains of MRL/*lpr* mice (42). MRL/*lpr* mice had more GFAP-positive astrocytes in the cornu ammonis 3 (CA3) region than MRL/*MPJ* mice (**Figures 1C, D**; P=0.009). In the dentate gyrus (DG) region, the number of GFAP-positive astrocytes did not significantly differ between the MRL/*lpr* and MRL/*MPJ* mice (**Figures 1E, F**; P=0.108). Senescent cells contribute to neuroinflammation (14–18, 43); therefore, we counted the number of senescent cells, using the marker SPiDER-β-Gal, in GFAP-positive astrocytes. We could not identify SPiDER-β-Gal–positive astrocytes in either MRL/*lpr* or MRL/*MPJ*. The number of Iba-1 positive microglia was higher in MRL/*lpr* mice than in MRL/*MPJ* mice in the CA3 region (**Figures 1G, H**; P = 0.033), but not in the DG region (**Figures 3C, D**; P = 0.429). Small numbers of SPiDER-β-Gal–positive microglia were present in both of MRL/*lpr* and MRL /*MPJ* mice; however, microglia were not the major population of SPiDER-β-Gal–positive senescent cells (**Figures 1I, J**).

### MRL/lpr Mice Have a Higher Proportion of Senescent NeuN+ Cells in CA3 Hippocampus

**Figure 2:**
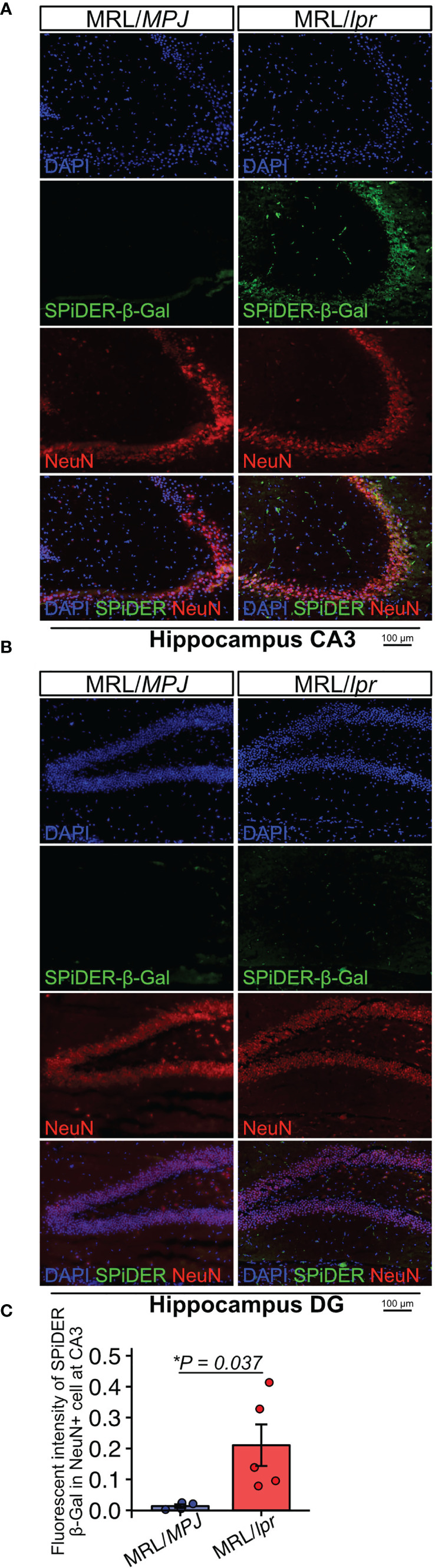
MRL/lpr mice have higher numbers of SPiDER-β-Gal– and NeuN-positive cells in the hippocampus. **(A, B)** Representative images of NeuN immunostaining and SPiDER-β-Gal staining of the hippocampus CA3 and DG regions in MRL/*MPJ* and MRL/*lpr* mice. **(C)** Quantitation of SPiDER-β-Gal intensity in NeuN-positive cells in MRL/*MPJ* and MRL/*lpr* mice. Quantitative data are shown as means ± SEs in dot plots. P-values were determined by two-tailed Student’s t-test. (*P < 0.05).

Next, to determine whether neurons exhibited features of senescence, we performed SPiDER-β-Gal staining and immunofluorescence with a NeuN antibody. SPiDER-β-Gal intensity in NeuN-positive cells was significantly higher in MRL/*lpr* mice than in MRL/MPJ mice in the hippocampus CA3 region (**Figures 2A**–**C**; P = 0.037). By contrast, in the DG region, SPiDER β-Gal expression was not detectable in either MRL/*lpr* or MRL/*MPJ* mice (**Figure 2B**).

### Neuro-2a Cells Induced to Senesce by Irradiation Exhibit a Neuroinflammatory Phenotype

**Figure 3:**
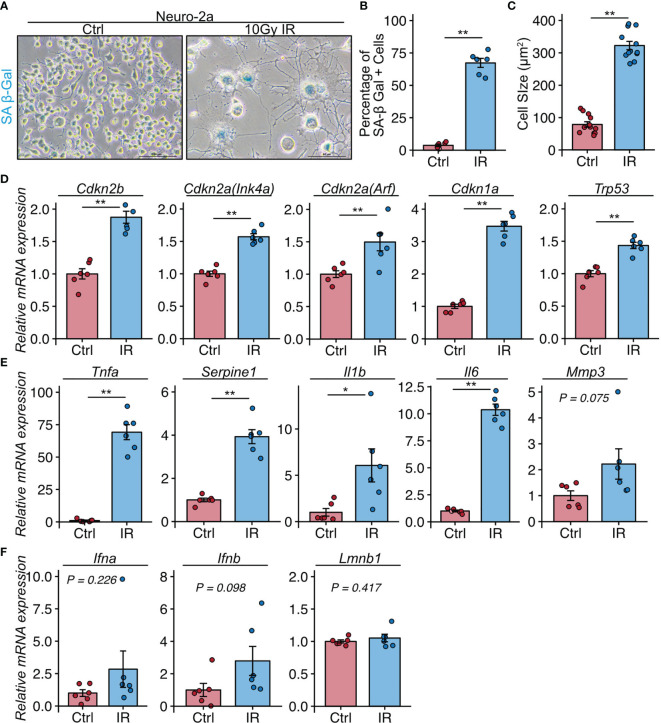
Neuro-2a cells induced to undergo senescence by irradiation exhibit a neuroinflammatory phenotype. **(A)** Representative images of SA-β-Gal expression after 10 Gy ionizing radiation (IR) in Neuro-2a cells in randomly chosen fields of view (n = 6 per group). **(B, C)** Quantitation of SA-β-Gal–positive cells and the cell size. **(D–F)** Relative mRNA expression of senescence and SASP-related genes in Neuro-2a cells with or without 10 Gy IR. Quantitative data are shown as means ± SEs in dot plots. P-values were determined by two-tailed Student’s t-test. (*P < 0.05, **P < 0.01).

These histological analyses indicated that neural cells in the CA3 region were a major population of senescent cells in lupus model mice with a depression-like phenotype. We next investigated whether senescent neural cells exhibited the features of cells that induce neuroinflammation in NPSLE. To induce senescence, we exposed Neuro-2a cells to 10 Gy irradiation and passaged them 3 days later to avoid the over-confluency that occurs post-irradiation due to Neuro-2a cell enlargement. Six days after irradiation, we harvested the cells and subjected them to SA-β-Gal staining and quantitative PCR analysis. Irradiated Neuro-2a cells exhibited senescent features including SA-β-Gal expression (**Figures 3A, B**), elevated cell size (**Figure 3C**), and upregulation of *Cdkn2a* (Ink4a and Arf), *Cdkn2b*, *Cdkn1a*, and *Trp53* (**Figure 3D**). The irradiated Neuro-2a cells also expressed high levels of genes encoded by SASP factors, including *Tnfa*, *Serpine1*, *Il6*, and *Il1b*, all of which are also upregulated in NPSLE (**Figures 3E, F**) (4).

### Fisetin Treatment Selectively Kills SPiDER-β-Gal–Positive Senescence Neural Cells *In Vitro*

**Figure 4:**
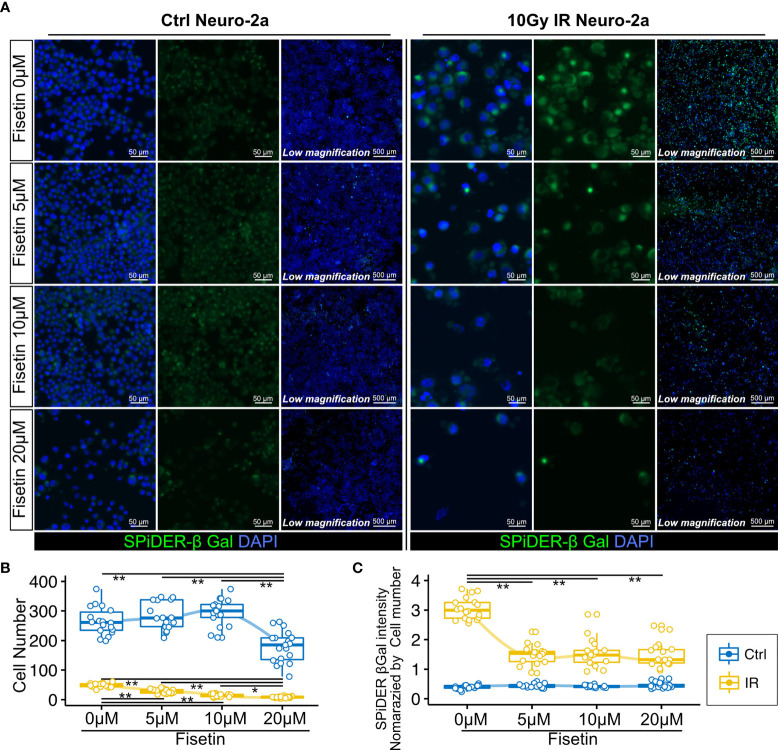
Effect of fisetin treatment on senescent Neuro-2a cells *in vitro*. **(A)** Representative images of SPiDER-β-Gal and F-actin staining in Neuro-2a cells with or without 10 Gy IR. **(B, C)** Quantitation of cell number and SPiDER-β-Gal intensity in Neuro-2a cells treated with the indicated concentrations of fisetin (0–20 µM) for 48 h. Quantitative data are shown as medians with IQRs and 1.5 times the IQR, and are displayed as dot plots and box-and-whisker plots. P-values were determined by one-way ANOVA adjusted by the Tukey method. P-values were determined by two-tailed Student’s t-test. (*P < 0.05 and **P < 0.01).

Next, we investigated whether the senolytic drug fisetin would selectively kill senescent neural cells. Fisetin, a flavonoid found in many fruits and vegetables, was previously identified as a senolytic compound (40). In addition, fisetin exhibits brain uptake potential and penetrates the blood–brain barrier more effectively than other flavonoids, including quercetin, luteolin, and myricetin (44–46). Hence, we used fisetin as a senolytic compound in this study. Unirradiated and irradiated Neuro-2a cells were treated with serial concentrations of fisetin (0–20 µM) for 48 h. The observed number of irradiated Neuro-2a cells significantly decreased in a dose-dependent manner (**Figures 4A, B**), and the number of control Neuro-2a cells significantly decreased after treatment with 20 µM fisetin (**Figures 4A, B**). SPiDER-β-Gal expression in irradiated Neuro-2a cells was significantly reduced at doses of 5, 10, and 20 µM (**Figure 4C**). Doses of 5 and 10 µM fisetin decreased the fraction of SPiDER-β-Gal–positive senescent Neuro-2a cells without affecting non-irradiated proliferating cells.

### Fisetin Treatment Reduced the Prevalence of the Depression-Like Phenotype and Number of Senescent Cells *In Vivo*

**Figure 5:**
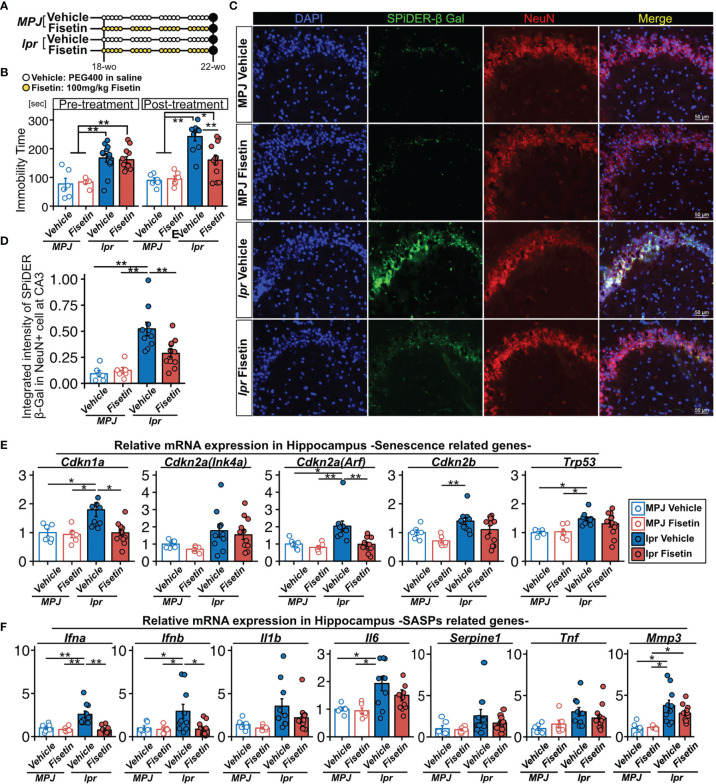
Effect of fisetin treatment on depression-like behavior in MRL/*lpr* mice. **(A)** Schematic diagram of the procedure for oral administration of fisetin to MRL/lpr and MRL/MPJ mice. **(B)** Quantification of the immobility time from the tail suspension test for MRL/*MPJ* and MRL/*lpr* mice with or without fisetin treatment. **(C)** Representative images of NeuN SPiDER-β-Gal immunostaining of the hippocampus CA3 regions and **(D)** the corresponding quantification of SPiDER-β-Gal intensity in NeuN-positive cells in MRL/*MPJ* and MRL/*lpr* mice with or without fisetin treatment. Relative mRNA transcription levels of **(E)** senescence- and **(F)** SASP-related genes in hippocampus isolated from MRL/*MPJ* and MRL/*lpr* mice with or without fisetin treatment. Quantitative data are shown as means ± SEs in dot plots. P-values were determined by two-tailed Student’s t-test. (*P < 0.05 and **P < 0.01).

To examine the senolytic effect of fisetin *in vivo*, we orally administered fisetin (100 mg/kg) or 20% PEG400 (as a control) to MRL/*lpr* (n=12 and 12, respectively) and MRL/MPJ (n=6 and 6, respectively) mice for 5 days every week for 4 weeks (**Figure 5A**). During this 4-week period, two MRL/lpr mice in the vehicle group died. Fifty percent of MRL/lpr mice die from renal failure by 24 weeks of age (47). After this 4-week period, we found that fisetin treatment reduced the prevalence of depression-like behavior in the MRL/*lpr* mice (**Figure 5B**). Fisetin treatment also reduced the observed SPiDER-β-Gal expression level in NeuN+ cells in the CA3 region (**Figures 5C, D**). PCR analysis showed that fisetin treatment reduced the mRNA transcription levels of *Cdkn1a* and *Cdkn2a(Arf)*, which are senescence factors, and *Ifna* and *Ifnb*, which are known SASP factors, in the hippocampus of the MRL/*lpr* mice (**Figures 5E, F**). In addition, vehicle-treated MRL/*lpr* mice exhibited significantly increased levels of Trp53, Il6, and Mmp3 mRNA transcription relative to the vehicle-treated MRL/MPJ mice, but no significant difference was observed for the fisetin-treated MRL/*lpr* mice relative to the vehicle-treated MRL/MPJ mice (**Figures 5E, F**).

## Discussion

Senescent cells limit their own proliferation but remain metabolically active, secreting a variety of factors including: inflammatory cytokines such as IL-6, IL-8, and TNF-⍺; chemokines; growth factors such as TGF-β; matrix metalloproteinases (MMPs); and micro-RNAs. Collectively, these secreted factors are referred to as the SASP (48). The SASP is considered a hallmark of cellular senescence when combined with other senescence markers such as the cytoplasmic marker SA-β-gal and the nuclear biomarkers p16^INK4a^, p21^WAF1/Cip1^, Ki67, and γH2AX (48–50). In this study, we show that MRL/lpr lupus-prone mice accumulate senescent NeuN-positive cells in the hippocampus. In addition, neural cells induced to undergo senescence increased mRNA expression of genes encoding SASP-related factors such as *Tnfa*, *Serpine1*, *Il6*, and *Il1b*, all of which are elevated in NPSLE (4). Because neurons are post-mitotic, non-cycling cells (those permanently in the G0 phase of the cell cycle), neuronal senescence, like that observed in other post-mitotic cells, relies on mechanisms other than proliferation arrest. Although it is not a fully specific marker, SA-β-Gal is considered to be a useful marker of cellular senescence, and the number of SA-β-Gal–positive neurons increases in aging mice and rats (51, 52). Furthermore, long-term culture–induced senescent neuronal cells exhibit elevated transcription levels of SASP genes (53). Several cytokines and chemokines were identified as biomarkers or therapeutic targets of NPSLE; in particular, type-1 interferons, TNFs, IL-6, and PAI-1, which are major components of the SASP, are present at elevated levels in the CSF of SLE patients (4, 6, 54, 55). Our results showed that the hippocampus isolated from MRL/*lpr* mice and irradiated senescent Neuro-2a cells exhibit upregulation of the transcription levels of SASP factors, supporting the idea that senescent neural cells contribute to the elevation of cytokines and chemokines in CSF.

In this study, the SA-β-Gal–positive senescent neural cells were observed in the CA3 region of the hippocampus, which is associated with depression (31–34). SLE and MRL/*lpr* mouse brain have an elevated population of damaged neural cells that express Fluoro Jade B (FJB) and also exhibit upregulation of ubiquitin in the CA3 region (56, 57). FJB dye is an anionic fluorescein derivative used for visualization of neuronal degeneration in brain tissue sections (58, 59), and ubiquitin binds to damaged or misfolded proteins (60). Most protein damage is not reversible, and degradation by the ubiquitin–proteasome system (UPS) eliminates damaged proteins (50, 61). Activation of the UPS is a key characteristic of the senescent state (50, 62).

We also demonstrated that fisetin exerts a potent senolytic effect in neural cells *in vivo* and *in vitro*. Several senolytic compounds have been reported, e.g., flavonoids, quercetin, curcumin, and luteolin (12, 13, 40). We used fisetin to target CNS senescence because it has higher brain uptake potential and more effective blood–brain barrier penetration than other flavonoids such as quercetin, luteolin, and myricetin (44–46). *In vivo*, fisetin treatment reduced the observed depression-like behavior in the mice and the number of senescent cells in the CA3 region of the hippocampus. In this study, fisetin treatment also reduced the transcription levels of several senescence- and SASP-related genes in the hippocampus. For example, the transcription level of the senescence gene *Cdkn1a* markedly increased in vehicle-treated MRL/*lpr* mice, and the level decreased after fisetin treatment. The number of p21-expressing NeuN-positive cells increases in older depressed patients relative to non-depressed older patients (63). In our study, type-I interferons, known SASP factors, are upregulated in MRL/lpr mice. Therapeutic administration of type-I interferons to mice with hepatitis C or other malignancies induces SLE-like psychiatric symptoms, including sickness behavior associated with depression, and inhibition of the type-I interferon receptor reduces anxiety-like behavior and cognitive deficits in lupus-prone mice (4, 54). If neural senescent cells produce type-I interferons, thereby exacerbating the development of NPSLE, senolytics targeting the causative cells may be effective treatments for NPSLE. IL-6 is a known pro-inflammatory SASP factor (8–13) that is upregulated in the hippocampus of MRL/lpr mice. The level of IL-6 observed in CFS is higher in NPSLE patients with an acute confusional state (ACS), which includes anxiety disorders, cognitive dysfunction, mood disorders, and psychosis, relative to those with diffuse NPSLE, states other than ACS, or those with focal NPSLE, which suggests that the IL-6 level observed in CFS may indicate the severity of NPSLE (64, 65). In this study, fisetin administration reduced the high transcription level of IL-6 in the hippocampus of MRL/lpr mice. Fisetin treatment causes a reduction of the transcription level of IL-6 in senescent cells in pulmonary fibrosis and aging-related pathology (40, 66). Although the varied and complex pathogenic pathways complicate the development of NPSLE therapies, our data indicate that fisetin treatment targeted specifically to NPSLE senescent neural cells results in inhibition of SASP factors such as type-I interferon and IL-6, suggesting that fisetin is a candidate NPSLE therapeutic. Fisetin not only has potential as a senolytic in neuronal cells, but also acts as a neuroprotective agent *via* its antioxidant, antitumor, anti-inflammatory, and anti-apoptosis effects (45, 67, 68). Hence, fisetin could be a candidate drug for CNS disorders by targeting neural cell populations. Our findings indicate that neural cells are a major population of senescent cells in the lupus-prone mouse model, whereas other studies reported that the major populations of senescent cells in Alzheimer’s model mice are astrocytes, microglia, and oligodendrocyte progenitor cells (17, 18). Furthermore, those studies used other senolytic compounds, dasatinib and quercetin (D+Q) and ABT263, to treat Alzheimer’s model mice, and administration of both senolytic compounds alleviated cognitive deficits and decreased the abundance of senescent cells in the brain (17, 18). Further study will be needed to identify the most effective senolytic compound for NPSLE.

In conclusion, our study highlights the accumulation of senescent neural cells in hippocampus of lupus-prone model mice. Oral administration of fisetin, a senolytic drug, reduced the number of senescent neural cells observed, the SASP expression level, and depressive behavior in MRL/lpr mice. These results indicate that the accumulation of senescent neural cells in the hippocampus plays a role in NPSLE pathogenesis, and therapies targeting senescent cells may represent candidate therapeutics for the treatment of NPSLE.

## Data Availability Statement 

The original contributions presented in the study are included in the article/**Supplementary Material**. Further inquiries can be directed to the corresponding author.

## Ethics Statement

The animal study was reviewed and approved by The Committee of the Animal Experimentation Center of the Sapporo Medical University School of Medicine.

## Author Contributions

YS: Conception and design, Collection and/or assembly of data, Manuscript writing, MM: Collection and/or assembly of data, SY: Data collection, TS: Data collection, NM: Data collection, MF: Manuscript writing, TSC: Conception and design, Collection and/or assembly of data, Manuscript writing, Final approval of manuscript. All authors contributed to the article and approved the submitted version.

## Funding

This study was supported by JSPS KAKENHI (Grant Number JP21H03049) and LEOC Co. Ltd. The funders had no role in study design, data collection and analysis, decision to publish, or preparation of the manuscript.

## Conflict of Interest

The authors declare that the research was conducted in the absence of any commercial or financial relationships that could be construed as a potential conflict of interest.

## Publisher’s Note

All claims expressed in this article are solely those of the authors and do not necessarily represent those of their affiliated organizations, or those of the publisher, the editors and the reviewers. Any product that may be evaluated in this article, or claim that may be made by its manufacturer, is not guaranteed or endorsed by the publisher.
